# Towards a model of GCN2 activation

**DOI:** 10.1042/BST20190331

**Published:** 2019-10-11

**Authors:** Glenn R. Masson

**Affiliations:** PNAC Division, MRC Laboratory of Molecular Biology, Francis Crick Avenue, Cambridge Biomedical Campus, Cambridge CB2 0QH, U.K.

**Keywords:** GCN2, ISR, P-stalk, protein translation, ribosome

## Abstract

Cells must be able to sense and adapt to their surroundings to thrive in a dynamic environment. Key to adapting to a low nutrient environment is the Integrated Stress Response (ISR), a short-lived pathway that allows cells to either regain cellular homeostasis or facilitate apoptosis during periods of stress. Central to the ISR is the protein kinase General Control Non-depressible 2 (GCN2), which is responsible for sensing starvation. Upon amino acid deficiency, GCN2 is activated and initiates the ISR by phosphorylating the translation initiation factor eIF2α, stalling protein translation, and activating the transcription factor ATF4, which in turn up-regulates autophagy and biosynthesis pathways. A key outstanding question is how GCN2 is activated from an autoinhibited state. Until recently, a model of activation focussed on the increase of deacylated tRNA associated with amino acid starvation, with deacylated tRNA binding directly to GCN2 and releasing autoinhibition. However, *in vivo* experiments have pointed towards an alternative, deacylated-tRNA-independent mechanism of activation. Here, we review the various factors that may facilitate GCN2 activation, including recent research showing that the P-stalk complex, a ribosome-associated heteropentameric protein complex, is a potent activator of GCN2.

## Introduction

Cells must be able to respond to stress in order to survive in a dynamic environment. These stresses can arise from intrinsic sources (such as the accumulation of misfolded proteins in the endoplasmic reticulum), extrinsic sources (such as hypoxia or amino acid deprivation), or from the advent of disease. One manner in which cells respond is the Integrated Stress Response (ISR) — an intricate, adaptive, short-lived, pro-survival pathway which is present in all eukaryotic organisms and allows a cell to both identify the loss of homeostasis and then alter its transcriptional and translational programs to rectify this imbalance [1,2]. The ISR achieves this by sensing stress and down-regulating global protein synthesis while simultaneously up-regulating both biosynthetic and autophagic pathways — the exact nature of the response is optimised to the particular stress. Under prolonged stress, the ISR may promote apoptosis, but induction of ISR is also crucial for cancers to survive and grow [3], making inhibition of ISR an attractive therapeutic strategy.

The ISR is initiated by four key stresses, each of which is sensed by an individual protein kinase — amino acid deprivation is sensed by the kinase GCN2 (general control non-depressible protein 2), heme deprivation by HRI (Heme-regulated eIF2α kinase), double-stranded RNA by PKR (double-stranded RNA-dependent protein kinase) and endoplasmic reticulum stress by PERK (PKR-like ER kinase). These four kinases all phosphorylate the same target, the eukaryotic translation initiation factor eIF2α, which, when phosphorylated, stalls cap-dependent mRNA translation and initiates a transcriptional stress response via the transcription factor ATF4. GCN2 has many roles not only in health but in disease also, including neurological disorders [4], pulmonary veno-occlusive disease [5], maintaining tumour growth and survival [3], and intracellular parasite replication [6] — providing fertile ground for further research in pharmaceutical sectors also.

How GCN2 is capable of recognising the state of amino acid deprivation has been a subject of investigation for some time, with much of this research has being conducted using *Saccharomyces cerevisiae* as a model organism. One outstanding question is whether the exact machinery and mechanism of GCN2 activation are wholly conserved between baker's yeast and higher eukaryotic organisms. A key consideration in the comparison between these systems is that *S. cerevisiae* contains only a single eIF2α kinase (GCN2), whilst higher eukaryotes have multiple eIF2α kinases as described above.

One proposed mechanism for GCN2 activation is dependent on the associated rise of deacylated tRNA molecules which occurs upon amino acid starvation [7]. Here, we discuss recent findings that describe additional mechanisms for GCN2 activation, such as direct stimulation of GCN2 kinase activity by the ribosomes via the P-stalk complex [8].

## Structure of GCN2 and mechanism of activation

**Figure 1.**
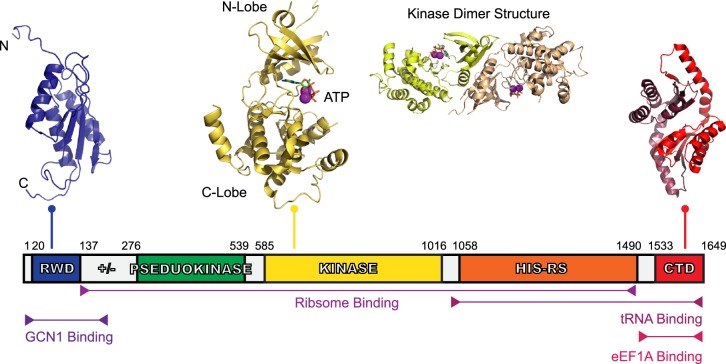
Domain organisation of human GCN2. GCN2 is a 1649 amino acid protein with five conserved domains: the RWD, the pseudokinase domain, kinase domain, HisRS-Like domain, and CTD. Linking the RWD and pseudokinase domain is ‘charged linker’ (labelled ‘+/−’) region also. The structure of GCN2's RWD domain from mouse was determined using nuclear magnetic resonance (NMR) spectroscopy [39], showing an α + β sandwich fold (PDB ID: 1UKX). The charged linker, a stretch of arginine, lysine, glutamate, and aspartate residues, precedes the pseudokinase domain — the structure of which has yet to be determined — and was identified as a pseudokinase domain due to the sequence similarity to typical kinase domains, however, it lacks key catalytic residues. The kinase domain, shows a typical kinase domain structure (PDB ID: 1ZYD) [10], and is presented as both a monomer and dimer with its associated ATP and Mg^2+^. Following the kinase domain is the HisRS-like domain, a domain essential for GCN2 activity *in vivo* [14] which interacts directly with tRNA [15]. Finally, there is the CTD, shown here using the dimeric mouse CTD structure (PDB ID: 4OTN) [27]. The CTD is constitutively dimeric, similar to the kinase domain. There is a species-dependent interaction of the CTD with ribosomes also, which is reliant on three lysine residues found in yeast. Yeast CTDs co-migrate with ribosomes on a sucrose gradient, but this is not the case with the mouse CTD where these lysine residues are absent.

The role of GCN2 as a nutrient sensor was determined as part of an initial screen of *S. cerevisiae* genes responsible for the general amino acid control (GAAC) pathway — a pathway similar to ISR [9]. Human GCN2 is 1649 amino acids long, with a molecular mass of ∼190 kDa (380 kDa as a dimer). The domain organisation of GCN2 provides insights into its mechanism of activation. GCN2 has five conserved folded domains (see Figure 1): an N-terminal RWD (RING-finger proteins, WD repeat-containing proteins and the yeast DEAD-like helicases) domain, a pseudokinase domain, a catalytically active kinase domain, a ‘HisRS-like’ domain (named due to sequence similarity to histidyl-tRNA synthetase) and a C-terminal domain (or CTD). In addition to this, there is also is a ‘charged linker’ region, a likely unstructured region found between the RWD and pseudokinase domains. Although a complete three-dimensional structure of GCN2 has yet to be determined, some of the isolated domains have been amenable to structural analysis (see Figure 1).

In a basal state (in non-starved cells), GCN2 forms an inactive homodimer, with multiple autoinhibitory interactions occurring between the CTD, the HisRS-like domains and the kinase domains preventing aberrant activation of the kinase [7]. The activation of GCN2 (upon amino acid starvation) is linked to a structural rearrangement and ultimately to a release of these multi-domain autoinhibitory contacts, while still maintaining a dimeric structure.

The crystal structure of the kinase domain of yeast GCN2 has been solved in both the active and inactive state [10], providing structural insight into how the enzyme may become activated. These data suggest that the kinase domain is inactive in the absence of any regulatory interactions and that the kinase domain maintains a ‘closed’ position due to the hinge between the N and C lobes being unusually rigid, drawing the lobes towards one another and partially occluding access to the catalytic pocket. In contrast, a constitutively active GCN2 mutant, R794G, shows a rearrangement of several key hinge residues, allowing the breakdown of a hydrogen-bonding network surrounding the hinge region. The resulting increase in hinge flexibility enables parting of the lobes and greater access to the GCN2 active site. Once opened, the kinase domain is free to autophosphorylate two threonine residues in the activation loop of the kinase domain, causing a stabilisation the active state [10]. Key to this activation is the generation of an intermolecular salt bridge between the kinase domains [7,11].

The network of inhibitory interactions that keeps GCN2 in the inactive dimeric state are proposed to involve the two N-Lobes of the kinase domain interacting with one another and with the HisRS-like domains [7]. In addition, the kinase domains may also be inhibited by interactions with the CTDs, with stabilisation provided by the two HisRS-like domains. This autoinhibitory network is disrupted upon GCN2 activation, allowing the formation of the stimulatory pseudokinase/kinase domain interactions that further enhance GCN2 activity. The current model for the trigger which initiates this reorganisation is that binding of deacylated tRNA (which accumulates upon amino acid starvation) to the HisRS-like domain of GCN2 causes a release of the associated CTDs and facilitates the interaction between the kinase and pseudokinase domains [10]. At least some of these autoinhibitory contacts are conserved in mouse and human GCN2, since the *in vitro* kinase activity of GCN2 is increased up to ∼20-fold when CTD is deleted [8,12]. It has also been shown that the charged linker region of GCN2, located between RWD and pseudokinase domains, played an additional, smaller role in inhibiting the enzyme, with its removal activating the enzyme 2-fold [8].

## Regulators of GCN2 activity: deacylated tRNA

One of the key regulators of GCN2 activity is deacylated tRNA. Under amino acid starvation, there is an increase in deacylated tRNA levels as tRNA synthetase enzymes fail to aminoacylate tRNA due to the low concentrations of amino acids. Microarray analysis of yeast cells has shown that there is a direct correlation between deacylated tRNA concentration and GCN2 activity [13]. GCN2 is capable of distinguishing between aminoacylated and deacylated tRNA species, and interactions between yeast GCN2 and deacylated tRNA have been demonstrated by both gel shift analysis and Northwestern assay and they are dependent on the HisRS-like domain of GCN2 [14].

Recently it has been shown through *in vitro* eIF2α kinase assays and surface-plasmon resonance studies, that total liver deacylated tRNA interacts with human GCN2 with an EC_50_ of ∼500 nM and a *K*_D_ of ∼2 µM (this was similar to yeast GCN2) [8]. Deacylated tRNA caused an ∼5- to 8-fold activation of GCN2, and mutation in the *m*2 motif — a motif found within the HisRS-like domain and previously shown to be key to GCN2/deacylated tRNA interaction [15] — ablated this interaction and activation by deacylated tRNA. Dimerisation of the HisRS-like domain is also crucial for tRNA binding [16], but in addition to the HisRS-like domain, the CTD of GCN2 also contributes to deacylated tRNA interaction. Three lysine residues found within the CTD (K1552, K1553, and K1556) are essential for both tRNA and ribosome binding [14]. The mechanism for how GCN2 is activated *in vivo* by tRNA *in vivo* may be dependent on two additional factors: GCN1 and GCN20.

## Regulators of GCN2 activity: GCN1 and GCN20

GCN1 (general control non-derepressible 1) is crucial to GCN2's activation in response to amino acid starvation *in vivo* [9,17] — yeast strains with a GCN1 knockout are unresponsive to amino acid starvation. GCN1 is not necessary, however, for GCN2's kinase activity nor deacylated tRNA-mediated stimulation *in vitro* [8], suggesting it has some sort of scaffolding or localisation role when found within a cellular environment. GCN1 is a large, 2671 amino acid protein, with sequence homology to the eukaryotic elongation factor 3 (eEF3), and a large HEAT repeat (a helical fold) section in the centre of the protein. Yeast GCN2 and GCN1 associate with one another *in vivo* [18] and also *in vitro* [19] — with the interface between the two proteins being composed of the RWD domain of GCN2 and the C-terminus of GCN1. The phosphorylation of eIF2α *in vivo* is impaired by overexpression of the RWD of GCN2 (as this sequesters GCN1 away from full-length GCN2) [20], and a single residue R2259 in yeast (R2312 in humans), found within the C-terminus of GCN1, is critical for this interaction also. GCN1 also has a strong association with ribosomes [20] and polysomes [21], with evidence from yeast two-hybrid studies that it binds to the ribosomal protein S10, found on the small subunit under the A-site [22].

General control non-derepressible 20 (GCN20) was identified alongside GCN1 and GCN2 [9] in initial yeast screens, but a mammalian homologue to GCN20 has yet to be identified, however. A possible candidate with partial sequence homology is ABC50 [23], but this fails to rescue functionality in GCN20 deficient cells. GCN20, similar to GCN1, shares sequence homology with the translation factor eEF3, and can also bind (albeit weakly) to polysomes [24], but it is very much smaller than either GCN1 or GCN2 at only 752 amino acids. GCN20 forms a stable complex with GCN1 via GCN20's N-terminus binding to the HEAT repeats of GCN1 [25]. GCN20 is a member of the ATP-binding cassette (ABC) family of proteins, with two ABC cassettes being present in the C-terminus of GCN20, sharing homology to eEF3.

This has lead to a hypothesis that the GCN1–GCN20 complex functions in a similar manner to eEF3, and facilitates the deacylated tRNA-mediated activation of GCN2. During normal protein translation, eEF3 promotes the release of the uncharged tRNA from the E-site of the ribosome*.* The hypothesis for GCN2 activation centres on the GCN1–GCN20 complex binding to the ribosome at the A-site via S10, and acting in a similar manner to eEF3 by ejecting deacylated tRNA, but from the A-site rather than E-site of the ribosome, and transferring it to GCN2 and relieving its autoinhibition [26]. The latest findings of the relatively low affinity of GCN2 for deacylated tRNA [when compared with the P-stalk (see below)] [8], could suggest that a concerted transfer event, or a high localised concentration of deacylated tRNA, may be necessary for GCN2 activation, which is not necessarily incompatible with this hypothetical mechanism of deacylated tRNA delivery to GCN2.

## Regulators of GCN2 activity: ribosomes and the ribosomal P-stalk

Investigations into GCN2's interaction and activation by the ribosome has presented a varied picture, and this may be due to potential differences between the mechanisms employed by yeast and higher eukaryotic pathways.

In a study which directly compared the two, yeast GCN2 co-migrated with translating ribosomes while mouse GCN2 did not co-migrate with ribosomes [27]. However, more recently, purified human GCN2 was shown to bind to rabbit ribosomes via pull-down assays [8]. Although the efficiency of the interaction was reduced when the CTD was removed from GCN2, the key requirements for GCN2 to interact with the ribosome were the three GCN2 ‘core’ domains — the pseudokinase, kinase, and HisRS-like domains. Importantly, it was shown that the ribosomes were capable of activating GCN2 very potently with EC_50_ ∼ 25 nM and a 20-fold increase in activity. Furthermore, no additional activation was observed on the addition of deacylated tRNA, suggesting that the ribosome and deacylated tRNA share a mechanism of GCN2 activation.

Deacylated tRNA independent mechanisms of GCN2 activation have been observed previously *in vivo*, and intriguingly, it centres on the propensity for ribosomes to stall under translational stress [28]. Ishimura et al. determined that GCN2 could be activated (both in terms of eIF2α phosphorylation levels and subsequent ATF4 activation) by translational misregulation in the absence of an increase in deacylated tRNA, suggesting an alternative mechanism, the ribosome-mediated activation mechanism.

Using hydrogen deuterium exchange mass spectrometry (HDX-MS), it has been possible to map where GCN2 was interacting with the ribosome [8]. GCN2 was observed to be interacting with the P-stalk, in particular with the domain II of the P-stalk protein uL10. The P-stalk is a flexible, lateral protuberance on the large ribosomal subunit, located above the A-site of the ribosome, and is a heteropentameric complex consisting of a single copy of uL10 protein (also known as P0), and two copies each of the proteins ‘P1’ and ‘P2’ [29]. The P-stalk is also a dynamic complex, with uL10 typically staying attached to the ribosome, and the P1 and P2 proteins existing in an equilibrium between a ribosome-bound state and free state within the cytoplasm. It also is an essential part of the ribosomal GTPase-associated center (GAC) that is involved in elongation factor recognition and in factor-dependent GTP hydrolysis. In addition, the P1/P2 proteins of the P-stalk play a key role in translational fidelity [30] — associating with the ribosome from the cytoplasm to ensure correct translation. The P-stalk also plays a key role in recruiting ribosome-inactivating factors to the ribosome [31]. All three P-stalks proteins have a highly conserved, 14-residues long C-terminal tail (with a consensus sequence SEESD(D/E)DMGFGLFD), and these tails serve as recognition elements for the recruitment of translation factors during polypeptide synthesis [32–36].

It has been recently shown that the isolated heteropentameric P-stalk can stimulate GCN2 activity *in vitro* in the absence of tRNA (EC_50_ ∼ 250 nM and ∼40-fold increase in activity of GCN2) [8]. It was further determined, through truncation analysis of individual uL10, P1 or P2 C-terminal tails, that with a greater the number of acidic C-terminal tails present in the P-stalk heteropentamer, there was a greater stimulation of GCN2 activity, and that effectively, the available number of C-terminal tails can tune the activity of GCN2 [8].

The free P1/P2 proteins of the P-stalk have been associated with GCN2 activation previously [12], and this was also shown to compete with deacylated tRNA. One crucial difference between these investigation was that Inglis et al. determined that uL10 was required for P1/P2 mediated activation, since uL10 domain II (insertion in the NTD of uL10), was a major site of interaction with GCN2. Consequently, both domain II of uL10 and the C-terminal tails of uL10, P1, and P2 are necessary for the maximal activation of GCN2. HDX-MS analysis of GCN2 highlighted structural rearrangements that occurred in GCN2 on the addition of the P-stalk, with changes in solvent exchange rates in the pseudokinase, kinase, and HisRS-like domains that could be due to the release of autoinhibitory contacts. The work of Jiménez-Díaz et al. was conducted both *in vivo* with *S. cerevisiae* and using a cell-free *in vitro* translation system. Interestingly, the P1/P2 proteins activated GCN2 when cells were placed under osmotic stress or in low glucose conditions, but the activation much less pronounced when cells were exposed to amino acid deprivation. Additionally Jiménez-Díaz et al. also determined in a cell-free *in vitro* translation system that P1/P2 proteins activated GCN2 specifically, and had no effect on the eIF2α kinases PKR and HRI.

These latest findings do not account for the possible roles that GCN1 and GCN20 may have in the activation of GCN2 and ribosomal binding. Both GCN1 and GCN20 are capable of interacting with the ribosome individually [20,24], and when present as a heterodimer (and in the presence of ATP), they have a much higher affinity for ribosomes [24]. GCN1's role in binding the ribosome may be key to the ISR *in vivo*. Mutation of two regions of GCN1 implicated in ribosome binding, ‘M7’ (residues 793–834 in human GCN1) and ‘M1’ (1508–1515) both decreased the association of GCN1 with ribosomes, and dampened eIF2α phosphorylation in response to amino acid starvation [21], suggesting that GCN1 is a key regulator of the GCN2/ribosome interaction *in vivo*.

## Towards a model of GCN2 activity

**Figure 2.**
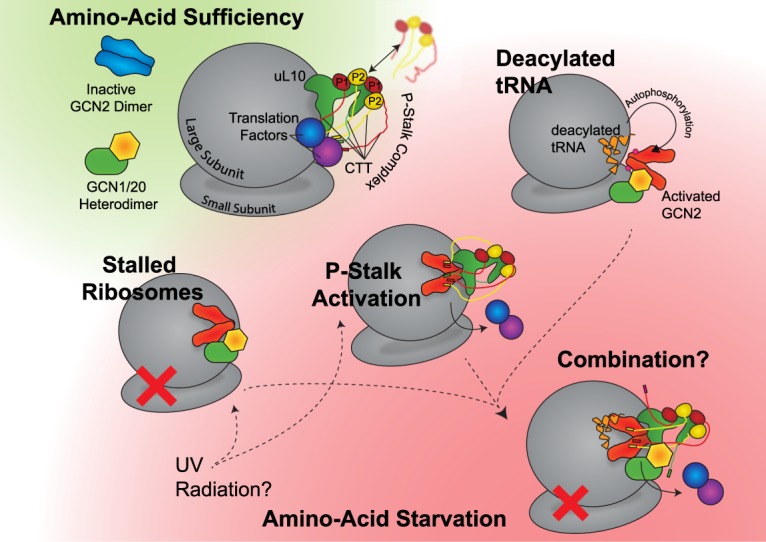
Potential routes to GCN2 activation. Whilst the autophosphorylated, activated state of GCN2 is thought to be common to all routes, it is not certain what pathways take precedence, whether they are mutually exclusive, whether they are present in all eukaryotic organisms, or whether they may be facilitated *in vivo* by GCN20 and GCN1. In non-starvation conditions, it is assumed that the P-stalk associates both the ribosome and translation factors around the A-site, whilst GCN1, GCN2, and GCN20 remain unbound to the ribosome in the cytosol. GCN2 remains autoinhibited, whilst GCN1 and GCN20 form a stable heterodimer. From this state, there are at least three possible routes to activation of GCN2 upon depletion of amino acids. Firstly in the case of stalled ribosomes; it may be that there is an increased affinity for the GCN1/2/20 complex for the stalled ribosome, causing an association and activation of GCN2. An alternate route to a stalled ribosome may be UV radiation. Secondly, upon starvation the P-stalk C-terminal tails may be free to bind to GCN2 due to the loss of translation factors — or alternative stresses (such as UV radiation) may also cause P1/P2 proteins to associate with uL10, forming the P-stalk and activating GCN2 in this manner. Thirdly, the increase in deacylated tRNA levels may cause an associated with GCN2, causing it to become activated in a manner that involves GCN1 and GCN20. Additionally, all three of these routes may combine, or be only partially correct.

Currently, building a model for GCN2 activation is fraught with difficulty (see Figure 2). Three key observations have been that (i) there are differences in the ribosome binding affinity between yeast and murine GCN2 [27], (ii) that there are tRNA-independent mechanisms for the activation of GCN2 [8,28], and (iii) ABC50, the apparent mammalian homologue of GCN20, does not rescue GAAC functionality in GCN20 deficient yeast cells [23]. From these observations it can be concluded that firstly, the sequence differences found in GCN2 between mice and yeast may be sufficient to manifest in differences in regulation, secondly, that deacylated tRNA may not be the sole activator of GCN2, and finally, that the regulatory machinery and mechanism that surrounds yeast GCN2 may not be conserved from yeast into higher Eukaryotes.

Given the wealth of convincing evidence for deacylated tRNA activating yeast GCN2, it certainly suggests that deacylated tRNA plays a key and potentially central role in activating GCN2 within that organism — but is deacylated tRNA the sole route to GCN2 activation, or are there multiple parallel pathways in higher Eukaryotes? Firstly, what are the roles of GCN1, GCN20, and the P-stalk within this dynamic? Do they facilitate deacylated tRNA-mediated activation, or are they autonomous entities that feed alternative information to GCN2, such as the available levels of elongation factors or ribosomal stalling status? It has been shown *in vitro* that deacylated tRNA, ribosomes, and the P-stalk are all capable of associating with and activating GCN2, all in the absence of GCN1 and GCN20. What are GCN1 and GCN20 contributing *in vivo*?

Although the ‘canonical’ stimulus to GCN2 activation focuses on amino acid deprivation, other stimuli exist, especially UV damage which has been reported in both fission yeast [17] and mammalian cells [37]. In their recent work, Anda et al. used fission yeast to determine that UV damage mediated GCN2 activation was dependent on GCN1, but it is unlikely that UV damage alters the levels of deacylated tRNA in the cell (indeed, UV damage is likely to reduce levels of translation globally [37]). Previously it has been suggested that there might be a UV-induced tRNA cross-linking event [26], where GCN2 becomes attached to tRNA, although this would presumably require GCN2 to be constantly nearby or bound to (deacylated) tRNA, in order for it to become cross-linked. Further attention should be given to determining how UV damage mediated GCN2 activation intersects with other activation mechanisms — especially in the context of the P-stalk, which responds to multiple cellular stresses by altering its association with the ribosome [38].

Key future experiments would be the determination of the structure of the complete GCN2 homodimer, both in an activated and inactivated state, and also the determination of ribosome-bound GCN2. In addition to this, deciphering the cellular localisation of GCN2 under both starvation and nutrient-abundant conditions may provide insight into its mechanism of activation. Perhaps the greatest insight into the mechanism of GCN2 activation could be provided from a ribosome-bound GCN2/1/20 heterotrimer structure. This may determine whether GCN1 and GCN20 are indeed acting in a manner similar to eEF3, and would provide context to relative roles of the P-stalk and deacylated tRNA.

**Perspectives**

- The activation GCN2 lies at the heart of the Integrated Stress Response (ISR), a key pathway that dictates how cells respond to detrimental conditions, such as those found within cancer tumours. Modulating the ISR is a target for pharmaceutical development, but currently, we have an incomplete understanding of how and when GCN2 may become activated.
- The greatest wealth of evidence for GCN2 activation stems from experiments in baker's yeast. In this system, upon amino-acid starvation the concentration of deacylated tRNA increases and binds to GCN2, activating it and initiating the ISR. Recent experiments, especially in higher eukaryotes, have pointed towards tRNA-independent methods of activation however, with the ribosome and the ribosomal P-stalk being potential mediators of GCN2 activation.
- Further experiments are required to determine how, where, and under what conditions GCN2 interacts with the ribosome, and whether this interaction is dependent on deacylated tRNA, or whether a certain ribosomal conformation is required. Additionally, the ostensible divergence in regulation between baker's yeast and higher eukaryotes requires clarity and warrants further investigation.

## Abbreviations

ABC: ATP-binding cassette
CTD: C-terminal domain
eEF3: eukaryotic elongation factor 3
GAAC: general amino acid control
GAC: GTPase-associated center
GCN1: general control non-derepressible 1
GCN2: general control non-depressible 2
GCN20: general control non-derepressible 20
HisRS: histidyl-tRNA synthetase
HRI: Heme-regulated eIF2α kinase
ISR: integrated stress response
NMR: nuclear magnetic resonance
PERK: PKR-like ER kinase
RWD: RING-finger proteins, WD repeat-containing proteins and the yeast DEAD-like helicases
